# Leistungsfähigkeit der McDonald-Kriterien von 2017

**DOI:** 10.1007/s00115-022-01410-2

**Published:** 2022-12-01

**Authors:** Franz Felix Konen, Philipp Schwenkenbecher, Mike P. Wattjes, Thomas Skripuletz

**Affiliations:** 1 Klinik für Neurologie mit Klinischer Neurophysiologie, Medizinische Hochschule Hannover, Carl-Neuberg-Str. 1, 30625 Hannover, Deutschland; 2 Institut für Diagnostische und Interventionelle Neuroradiologie, Medizinische Hochschule Hannover, Hannover, Deutschland

**Keywords:** Multiple Sklerose, Diagnosekriterien, Oligoklonale Banden, Liquor, Review, Multiple sclerosis, Diagnostic criteria, Oligoclonal bands, Cerebrospinal fluid, Review

## Abstract

**Hintergrund:**

Die schnelle und zuverlässige Diagnose einer Multiplen Sklerose (MS) ist entscheidend, um eine angepasste verlaufsmodifizierende Therapie zu beginnen. Die 2017-Revision der McDonald-Kriterien hat das Ziel, eine einfachere und frühzeitigere MS-Diagnose mit hoher diagnostischer Genauigkeit zu ermöglichen.

**Ziel der Arbeit/Fragestellung:**

In der vorliegenden Arbeit wurden die publizierten Arbeiten, die die Anwendung der McDonald-Kriterien von 2017 und 2010 miteinander verglichen haben, ausgewertet und bezüglich der diagnostischen Leistungsfähigkeit analysiert.

**Material und Methoden:**

Mittels Literaturrecherche in der PubMed-Datenbank (Suchbegriff: McDonald criteria 2010 and McDonald criteria 2017) wurden 20 Studien und ein Übersichtsartikel mit insgesamt 3006 auswertbaren Patienten identifiziert.

**Ergebnisse:**

Bei Anwendung der McDonald-Kriterien von 2017 konnte die Diagnose einer MS bei mehr Patienten (2277/3006 Patienten, 76 %) und in einem früheren Stadium (3–10 Monate) verglichen mit der Revision von 2010 (1562/3006 Patienten, 52 %) gestellt werden. Von den zusätzlichen MS-Diagnosen sind 193/715 auf die Anpassung der bildgebenden Kriterien der zeitlichen Dissemination und 536/715 auf die Einführung der oligoklonalen Banden als diagnostisches Kriterium zurückführen.

**Diskussion:**

Die revidierten McDonald-Kriterien von 2017 erlauben die Diagnosestellung einer MS bei einem höheren Anteil an Patienten beim ersten klinischen Ereignis.

## Abstract (en)

**Background:**

A rapid and reliable diagnosis of multiple sclerosis (MS) is crucial to initiate adapted disease-modifying treatment. The 2017 McDonald criteria were revised with the aim of further improving the diagnostic performance.

**Objective:**

In this article the published studies comparing the use of the 2017 and 2010 McDonald criteria were reviewed and analyzed in terms of diagnostic performance.

**Material and methods:**

A total of 20 studies and 1 review article with a total of 3006 evaluated patients were identified by means of a literature search in the PubMed database (search term: McDonald criteria 2010 and McDonald criteria 2017).

**Results:**

Using the 2017 McDonald criteria, a diagnosis of MS was made in more patients (2277/3006 patients, 76%) and in an earlier stage (3–10 months) compared with the 2010 revision (1562/3006 patients, 52%). Of the additional MS diagnoses, 193/715 were due to the adjustment of the imaging criteria of temporal dissemination and 536/715 were due to the introduction of oligoclonal bands as a diagnostic criterion.

**Conclusion:**

The revised McDonald criteria of 2017 have achieved their goal and enable the diagnosis of MS in a higher proportion of patients at the first clinical event.

## Hintergrund

Ziel der 2017-Revision der McDonald-Kriterien ist die schnellere und einfachere Diagnosestellung einer Multiplen Sklerose (MS) mit hoher diagnostischer Genauigkeit [28]. Hierfür wurden die Kriterien der zeitlichen und räumlichen Dissemination für die Magnetresonanztomographie (MRT) angepasst und der Nachweis liquorspezifischer oligoklonaler Banden (OKB) als zusätzliches Kriterium zur Erfüllung der zeitlichen Dissemination festgelegt [28]. Bisher verglichen 20 Studien und ein Übersichtsartikel die diagnostische Leistungsfähigkeit der McDonald-Kriterien von 2010 und 2017 in der Diagnose der MS [19, 22, 28].

## Material und Methoden

Für die Literaturrecherche wurde die NIH National Library of Medine PubMed.gov Datenbank (https://pubmed.ncbi.nlm.nih.gov/) genutzt. Mit dem Suchbegriff „McDonald criteria 2010 and McDonald criteria 2017“ wurden insgesamt 76 Artikel identifiziert. Diese Artikel wurden dahingehend bewertet, ob die diagnostische Wertigkeit der 2010- bzw. 2017-Revision der McDonald-Diagnosekriterien für MS miteinander verglichen wurde. Insgesamt wurden 15 Originalartikel und 5 „letter“ bzw. „short communications“ sowie ein Übersichtsartikel der englischsprachigen Literatur identifiziert und berücksichtigt (Tab. 1). Die Neuerungen der McDonald-Kriterien von 2017 sowie eine vereinfachte Zusammenfassung der Diagnosestellung einer MS anhand der revidierten Kriterien sind in Tab. 2 aufgeführt.

**Table 1:** 

Autoren	Einschlusskriterien	N	Alter	F/M-Ratio	Follow-up (Range)	Zusätzliche MS-Diagnosen basierend auf Kriterien der zeitlichen Dissemination (2017)		Sensitivität	Spezifität
						Liquorspezifische OKB	Symptomatische MRT-Läsionen		
Banerjee et al. 2019 [1]	KIS (gemäß McDonald-Kriterien 2010)	82	34,9^a^	1,3	29,5 Monate (24–84)^b^	NV	NV	2017: 53 %	2017: 79 %
								2010: NV	2010: NV
Beesly et al. 2018 [2]	KIS (gemäß McDonald-Kriterien 2010)	102	36^a^	2	4,3 Jahre (2,0–7,3)^a^	24/88 (27 %)	16/88 (18 %)	2017: 86 %	NV
								2010: 81 %	
Fadda et al. 2018 [4]	Erstes klinisches Schubereignis verdächtig für MS	71	14^b^	2,6	76 Monate (38–111)^b^	NV	NV	2017: 71 %	2017: 95 %
								2010: 53 %	2010: 97 %
Filippi et al. 2022 [5]	KIS (Einschlusszeitraum 1995 bis 2020, Angabe der zugrunde liegenden diagnostischen Kriterien NV)	785	32^b^	2,1	69,1 Monate (39,8–112,1)^b^	NV	NV	2017: 82 %	2017: 46 %
								2010: 65 %	2010: 66 %
Gaetani et al. 2018 [6]	KIS (gemäß McDonald-Kriterien 2010) + räumliche Dissemination (MRT)	137	31,4^a^	2,9	3,8 Jahre (±2,9)^a^	105/137 (77 %)	58/137 (42 %)	2017: 83 %	NV
								2010: 71 %	
Gobbin et al. 2019 [7]	Erstes klinisches Schubereignis verdächtig für MS	55	30^b^	1,5	51 Monate (38–81)^b^	4/5 (80 %)	1/5 (20 %)	2017: 100 %	2017: 14 %
								2010: 81 %	2010: 45 %
Habek et al. 2018 [8]	KIS (gemäß McDonald-Kriterien 2010)	113	32,1^a^	0,4	24 Monate^a^	43/44 (98 %)	15/44 (34 %)	2017: 85 %	2017: 63 %
								2010: 41 %	2010: 85 %
Hacohen et al. 2020 [9]	Erstes klinisches Schubereignis verdächtig für MS	156	13,7^b^	2,5	50,4 Monate (31,75–66,25)^b^	35/35 (100 %)	3/35 (9 %)	2017: 84 %	2017: 92 %
								2010: 47 %	2010: 97 %
Hyun et al. 2019 [10]	KIS (gemäß McDonald-Kriterien 2010)	163	31^a^	2,1	63 Monate (5–170)^a^	NV	NV	2017: 89 %	2017: 43 %
								2010: 53 %	2010: 69 %
Lee et al. 2019 [12]	Erstes klinisches Schubereignis verdächtig für MS + räumliche Dissemination (MRT)	290	36,4^a^	2,1	2,2 Jahre (±1,3)^a^	121/121 (100 %)	10/121 (8 %)	2017: 100 %	2017: 59 %
								2010: NV	2010: NV
Mantero et al. 2018 [13]	Erstes klinisches Schubereignis verdächtig für MS	3	24,7^a^	2	NV	1	1	2017: 2/3	NV
								2010: 0/3	
McNicholas et al. 2019 [15]	KIS (gemäß McDonald-Kriterien 2010, mit Konversion zu MS)	267	33^a^	2,6	4,6 Jahre (±2)^a^	127/200 (64 %)	13/125 (10 %)	2017: 44 %	NV
								2010: 16 %	
Pagani Cassara et al. 2020 [16]	Erstes klinisches Schubereignis verdächtig für MS	108	32,3^a^	2,3	5,96 Jahre (2–19)^a^	34/36 (94 %)	13/36 (36 %)	2017: 68 %	2017: 54 %
								2010: 31 %	2010: 79 %
Rojas et al. 2021 [20]	KIS (Einschlusszeitraum 1983–2019, Angabe der zugrunde liegenden diagnostischen Kriterien NV)	1188	39,8^a^	3,1	NV	NV	NV	NV	NV
Schwenkenbecher et al. 2018 [21]	Erstes klinisches Schubereignis verdächtig für MS	325	34,2^a^	1,8	47 Monate (24–87)^b^	76/78 (97 %)	16/78 (21 %)	2017: 66 %	NV
								2010: 42 %	
Souissi et al. 2020 [25]	KIS (Einschlusszeitraum 2003–2018, Angabe der zugrunde liegenden diagnostischen Kriterien NV)	98	35^a^	3,9	5 Jahre (±4)^a^	18/32 (56 %)	15/32 (47 %)	2017: 77 %	2017: 33 %
								2010: 44 %	2010: 63 %
Tintore et al. 2021 [27]	KIS (Einschlusszeitraum 1994–2020, Angabe der zugrunde liegenden diagnostischen Kriterien NV)	1174	31,6^b^	2,1	9,1 Jahre (0,01–25,2)^b^	NV	NV	2017: 55 %	NV
								2010: 45 %	
Van der Vuurst de Vries et al. 2018 [29]	Erstes klinisches Schubereignis verdächtig für MS	229	33,5^a^	2,7	65,3 Monate (±30,9)^a^	32/51 (63 %)	19/51 (37 %)	2017: 68 %	2017: 61 %
								2010: 36 %	2010: 85 %
Wong et al. 2018 [31]	Erstes klinisches Schubereignis verdächtig für MS	110	14,8^b^	1,7	4,5 Jahre (2,6–6,6)^b^	14/21 (67 %)	7/21 (33 %)	2017: 83 %	2017: 73 %
								2010: 49 %	2010: 87 %
Zheng et al. 2020 [33]	Erstes klinisches Schubereignis verdächtig für MS	93	37^b^	1,6	44 Monate (29–62,5)^b^	27/38 (71 %)	16/38 (42 %)	2017: 75 %	2017: 47 %
								2010: 15 %	2010: 100 %

Abweichende Zahlenwerte ergeben sich durch die unvollständige Verfügbarkeit von Magnetresonanztomographie(MRT)-Untersuchungen mit Kontrastmittelapplikation oder Liquoranalysen einschließlich Bestimmung der oligoklonalen Banden (OKB)

*N* Anzahl eingeschlossener Patienten, *Alter* Patientenalter in Jahren, *F/M-Ratio* Frauen/Männer-Ratio, *MS* Multiple Sklerose, *KIS* klinisch isoliertes Syndrom, *OKB* oligoklonale Banden, *NV* nicht vorhanden

^a^Mittelwert

^b^Median

**Table 2:** 

Kriterium der McDonald-Kriterien	Neuerung der Revision von 2017
Räumliche Dissemination	Keine Unterscheidung zwischen symptomatischen und asymptomatischen MRT-Läsionen
Räumliche Dissemination	Kortikale MRT-Läsionen gelten als äquivalent zu juxtakortikalen MRT-Läsionen
Zeitliche Dissemination	Keine Unterscheidung zwischen symptomatischen und asymptomatischen MRT-Läsionen
Zeitliche Dissemination	Nachweis oligoklonaler Banden im Liquor als zusätzliches diagnostisches Kriterium
Erfüllung der räumlichen + zeitlichen Dissemination der Revision von 2017 (definitive Multiple Sklerose)	Mind. 2 klinische Schübe^a^ + mind. 2 MRT-Läsionen^b^
Erfüllung der räumlichen + zeitlichen Dissemination der Revision von 2017 (definitive Multiple Sklerose)	Mind. 2 klinische Schübe + 1 MRT-Läsion^c^
Erfüllung der räumlichen + zeitlichen Dissemination der Revision von 2017 (definitive Multiple Sklerose)	1 klinischer Schub + mind. 2 MRT-Läsionen^b^ + mind. 1 kontrastmittelaffine MRT-Läsion/neue Läsionen in Verlaufs-MRT-Untersuchungen/oligoklonale Banden im Liquorbefund

*MS* Multiple Sklerose, *MRT* Magnetresonanztomographie

^a^Schub = monophasisches klinisches Ereignis mit unifokaler oder multifokaler Symptomatik, welche eine entzündliche Demyelenisierung widerspiegelt, subakut bis akut beginnt, für mindestens 24 h anhält, sich teilweise oder vollständig zurückbildet und nicht in Assoziation zu erhöhter Körpertemperatur oder Infektion auftritt

^b^Entzündungsherde in 2 der 4 MS-typischen neuroanatomischen Areale

^c^Klinische Symptomatik des Schubes sollte einen Entzündungsherd in einem anderen neuroanatomischen Areal vermuten lassen

Für die statistische Auswertung der aus den eingeschlossenen Artikeln entnommenen Werte für Sensitivität und Spezifität sowie die grafische Darstellung wurde GraphPad Prism (La Jolla, CA, USA; Version 5.02) verwendet. Die Zahlenwerte wurden, wenn nicht anders angegeben, als Mittelwert inklusive des 95 %-Konfidenzintervalls (KI) angegeben. Mittels Shapiro-Wilk-Tests wurde auf Normalverteilung überprüft. *p*-Werte < 0,05 wurden als statistisch signifikant angesehen. Für den Gruppenvergleich normalverteilter Werte wurde der Unpaired t‑Test verwendet, für nichtnormalverteilte der Mann-Whitney-U-Test.

## Ergebnisse

### Studienpopulationen

Verschiedene Studien untersuchten in retro- und prospektiven Analysen den Einfluss der 2017-McDonald-Kriterien im Vergleich zur Revision von 2010 (Tab. 1). 17 Studien untersuchten erwachsene Patienten, die sich mit dem ersten klinischen Ereignis vereinbar mit einem MS-Schub oder mit einem klinisch isoliertem Syndrom (KIS), teilweise mit Erfüllung des Kriteriums der räumlichen Dissemination, vorstellten [1, 2, 6–8, 10, 12, 13, 15, 16, 21, 25, 27, 29, 33]. Das mittlere Alter der eingeschlossenen Patienten lag zwischen 24,7 und 39,1 Jahren, die Frauen/Männer-Ratio lag zwischen 0,4 und 3,9 [1, 2, 6–8, 10, 12, 13, 15, 16, 21, 25, 27, 29, 33]. Weiterhin wurden in 3 Studien Kinder untersucht, welche sich mit dem ersten klinischen Ereignis vorstellten [4, 9, 31]. Bei diesen lag das Alter im Mittel bei 14,2 Jahren und die Frauen/Männer-Ratio bei 2,3 [4, 9, 31]. Das gepoolte Follow-up-Intervall aller Studien lag bei 53 Monaten (KI 45, 65; [1, 2, 4–10, 12, 13, 15, 16, 21, 25, 27, 29, 31, 33]). Insgesamt wurden Studienpopulationen aus 29 verschiedenen Nationen berücksichtigt.

### Zusätzliche MS-Diagnosen

Ein exakter Vergleich der verschiedenen Studien ist methodisch schwierig, da die Datenlage unterschiedliche Studiendesigns, Einschlusskriterien, Studienpopulationen und Follow-up-Intervalle beinhaltet (Tab. 1). Diese Faktoren beeinflussen maßgebend das Ergebnis der jeweiligen Studie, sodass im Folgenden zusätzlich die 95 %-Konfidenzintervalle (KI) angegeben werden.

**Figure 1:**
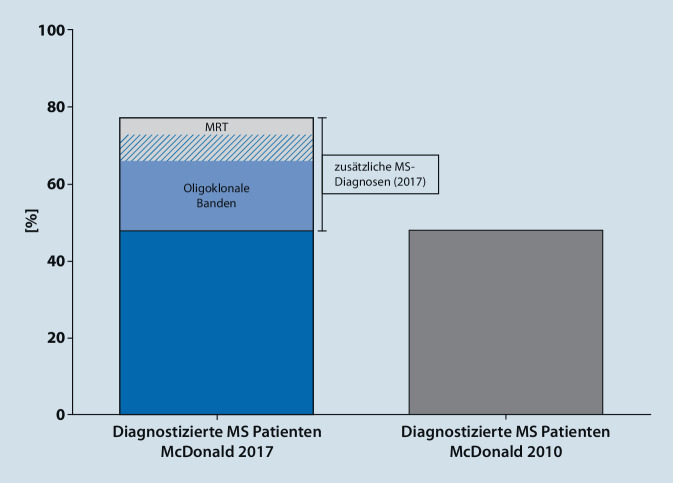

Unter Berücksichtigung aller vollständigen Datensätze konnte anhand der McDonald-Kriterien von 2010 die Diagnose einer MS bei 1562/3006 (52 %) aller eingeschlossenen Patienten gestellt werden, während eine MS gemäß den McDonald-Kriterien von 2017 bei 2277/3006 (76 %) Patienten diagnostiziert wurde (Abb. 1; [1, 2, 4–10, 12, 13, 15, 16, 21, 25, 27, 29, 31, 33]). Weiterhin verglichen einige der Studien die jeweilige Sensitivität der Kriterien der zeitlichen und räumlichen Dissemination der Revisionen von 2010 und 2017. Das Kriterium der zeitlichen Dissemination der McDonald-Kriterien von 2010 wies eine mittlere diagnostische Sensitivität von 47 % (KI 28 %, 67 %) bei MS-Patienten auf, während diese beim Kriterium der räumlichen Dissemination bei 76 % (KI 66 %, 86 %) lag [1, 2, 4–10, 12, 13, 15, 16, 21, 25, 27, 29, 31, 33]. Im Gegensatz hierzu war die mittlere diagnostische Sensitivität sowohl des Kriteriums der räumlichen (85 %; KI 79 %, 92 %), als auch der zeitlichen Dissemination (85 %; KI 79 %, 90 %) der Revision von 2017 höher und erreichte für die zeitliche Dissemination statistische Signifikanz (*p* = 0,0007; [1, 2, 4–10, 12, 13, 15, 16, 21, 25, 27, 29, 31, 33]).

### Einfluss der revidierten McDonald-Kriterien

Die gepoolte diagnostische Sensitivität der revidierten McDonald-Kriterien von 2010 der vorliegenden Studien beträgt 49 % (KI 38 %, 59 %), während die der McDonald-Kriterien von 2017 signifikant höher (*p* < 0,0001) ist und im Mittel 77 % (KI 70 %, 84 %) beträgt. Studienübergreifend wurde eine niedrigere diagnostische Spezifität der revidierten McDonald-Kriterien von 2017 (59 %; KI 44 %, 74 %) verglichen mit den Kriterien von 2010 (81 %; KI 68 %, 93 %; *p* = 0,0284) berichtet [1, 2, 4–10, 12, 13, 15, 16, 21, 25, 27, 29, 31, 33].

## Diskussion

Nach aktueller Studienlage ermöglicht die Revision der McDonald-Kriterien von 2017 eine frühere Diagnosestellung der MS und somit einen früheren Therapiebeginn. Im Vergleich zur vorangegangenen 2010-Revision der McDonald-Kriterien führt die Anwendung der aktuellen Revision zu einem höheren Anteil an Patienten, welche zum Zeitpunkt des ersten klinischen Schubereignisses die Diagnose einer MS erhalten [26]. Weiterhin konnte gezeigt werden, dass die Anwendung der neuen McDonald-Kriterien zu einer Verkürzung des Zeitraums zwischen erstem klinischen Schubereignis und definitiver MS-Diagnose geführt hat. So wurde berichtet, dass die MS-Diagnosestellung nach den McDonald-Kriterien von 2017 um 3 bis 10 Monate im Vergleich zur Revision von 2010 antizipiert werden konnte [1, 2, 4–10, 12, 13, 15, 16, 21, 25, 27, 29, 31, 33]. Rojas et al. und Tintore et al. konnten zeigen, dass sich der Trend zu einer immer früheren Diagnosestellung einer MS bzw. zu einer Verkürzung des Zeitraums zwischen erstem klinischen Schubereignis und definitiver MS-Diagnose über frühere Revisionen der McDonald-Kriterien bis hin zur aktuellen von 2017 fortsetzte [21, 27]. Weiterhin zeigt die Studie von Tintore et al., dass das Ziel, eine effektive Therapie frühestmöglich zu beginnen, ebenfalls erreicht wurde [27]. Auch diesbezüglich zeichnet sich ein Trend früherer Diagnosekriterien zu den aktuell revidierten von 2017 ab [27].

Insgesamt führte die Revision der McDonald-Kriterien von 2017 zu einer Vereinfachung der diagnostischen Kriterien, da nicht mehr zwischen symptomatischen und asymptomatischen MRT-Läsionen bzw. kortikalen und juxtakortikalen Lokalisationen unterschieden wird [26, 28]. Im Mittel sind 27 % (KI 19 %, 34 %) der zusätzlichen MS-Diagnosen nach den McDonald-Kriterien von 2017 auf die Veränderung der MRT-Kriterien der zeitlichen Dissemination zurückzuführen (keine Unterscheidung zwischen symptomatischen und asymptomatischen Entzündungsherden; [1, 2, 4–10, 12, 13, 15, 16, 21, 25, 27, 29, 31, 33]). Im Gegensatz hierzu beträgt dieser Anteil beruhend auf der Einführung liquorspezifischer oligoklonaler Banden als zusätzliches Kriterium der zeitlichen Dissemination im Mittel 75 % (KI 62 %, 88 %; [1, 2, 4–10, 12, 13, 15, 16, 21, 25, 27, 29, 31, 33]). Hierbei gilt es zu berücksichtigen, dass in den eingeschlossenen Studien zumeist nicht näher unterschieden wurde, ob die zusätzlichen MS-Diagnosen anhand des Nachweises oligoklonaler Banden oder der MRT-Bildgebung erfolgte. Der Einschluss des Nachweises der oligoklonalen Banden als zusätzliches Kriterium zur Erfüllung der zeitlichen Dissemination in die revidierten McDonald-Kriterien von 2017 hat zwar zu einer schnelleren MS-Diagnose geführt. Es gilt jedoch zu berücksichtigen, dass oligoklonale Banden trotz der sehr hohen Prävalenz bei MS nicht spezifisch für eine MS sind [32]. Im Gegenteil weisen sie eine intrathekale IgG-Synthese nach, welche auch bei anderen autoimmunen oder infektiösen ZNS-Erkrankungen oder neurologisch gesunden Patienten beobachtet werden kann [17, 32]. Vor diesem Hintergrund ist es wichtig zu berücksichtigen, dass die erhöhte Sensitivität der aktuellen Revision der McDonald-Kriterien die Gefahr falscher MS-Diagnosen birgt [3, 23, 24]. Vorsicht ist bei Patienten geboten, welche formell radiologisch die Diagnosekriterien erfüllen, sich aber mit untypischen klinischen Symptomen vorstellen [3, 23, 24, 28]. Da die McDonald-Kriterien bei Patienten angewandt werden sollen, die sich mit einem „typischen“ erstmaligen inflammatorsich-demyelinisierenden Ereignis vorstellen, erfordert die Diagnosestellung einer MS und die Abgrenzung von verschiedenen Differenzialdiagnosen besondere Expertise [3, 23, 24, 28]. Als „typische“ demyelinisierende Syndrome sind hierbei unilaterale Optikusneuritiden, internukleäre Ophthalmoplegien, gesichtsbetonte Hypästhesien, zerebelläre Syndrome (Ataxie, Nystagmen) und Beeinträchtigungen der Sensomotorik, welche einer Schädigung des Rückenmarks zuzuordnen sind, anzusehen [24, 28]. Im Gegenteil weisen „red flags“ wie (bilaterale) schwere, nichtremittierende Optikusneuritiden, extensive langstreckige Myelitiden und Area-postrema-Syndrome wie Schluckauf oder Erbrechen auf andere differenzialdiagnostisch zu bedenkende demyelinisierende Syndrome hin [18, 24]. Klinische Symptomatik und Ergebnisse der MRT-Bildgebung können auch bei Patienten mit Migräne und kardiovaskulären (insbesondere mikrovaskulären) Komorbiditäten fälschlicherweise den Verdacht auf eine MS lenken [3, 23, 24]. Insbesondere in diesen Fällen gilt es, das typische perivaskuläre Verteilungsmuster von MS-assoziierten MRT-Läsionen zu beachten [23, 24]. Auch wenn die aktuelle Revision der McDonald-Kriterien keine distinkte Anzahl oder Konfiguration der Läsionen zur Stellung der MS-Diagnose fordert, lassen sich MS-Fehldiagnosen vermeiden, wenn in diesen Patientenkollektiven mit Läsionen der weißen Substanz mindestens drei periventrikuläre Läsionen oder größere Läsionen zur Darstellung kommen [24]. Zur weiteren Erhöhung der Spezifität bei der Interpretation auffälliger MRT-Befunde gilt es zu berücksichtigen, dass punktförmige Läsionen eine Größe von mindestens 3 mm im Durchmesser (oft sogar größer als 6 mm) aufweisen sollten und dass juxtakortikale und periventrikuläre Läsionen dem Kortex bzw. dem Seitenventrikel anliegen, ohne zwischenliegende normale weiße Substanz [24]. Insbesondere die fehlerhafte Interpretation einzelner, sehr kleiner Läsionen mit ausgeprägtem Kontakt zur weißen Substanz trägt zur Fehldiagnose einer MS bei [24]. Weiterhin lässt sich die MS-Diagnose sichern, wenn Entzündungsherde in MS-typischen neuroanatomischen Arealen (insbesondere auch spinal) in wenigstens zwei verschiedenen MRT-Sequenzen zu identifizieren sind [24, 28]. Doch auch bei der Präsentation mit kontrastmittelaffinen spinalen Läsionen ist Vorsicht geboten, da beim Vorliegen eines weiteren zerebralen Entzündungsherdes die Stellung der MS-Diagnose bereits möglich ist [28]. Da diese Läsionen jedoch nicht spezifisch für eine MS sind, sondern im Gegenteil sogar bei einer großen Anzahl andersartiger inflammatorischer oder infektiöser Differenzialdiagnosen vorliegen können, ist hier die Gefahr einer fehlerhaften MS-Diagnose gegeben [3, 23, 24]. Dies unterstreicht die dringende Notwendigkeit einer standardisierten MRT-Akquisition des Gehirns und des Rückenmarks sowie die standardisierte Interpretation und Befundung des Bildmaterials entsprechend internationalen Expertenkonsensusempfehlungen [30]. Zusätzlich lässt der Nachweis oligoklonaler Banden eine MS-Diagnose wahrscheinlicher erscheinen [11, 14]. Lassen sich initial keine oligoklonalen Banden nachweisen, sollte im Verlauf eine erneute Liquoranalyse durchgeführt werden, um eine Konversion des Status oligoklonaler Banden aufzuzeigen, insbesondere bei KIS-Patienten mit hohem Risiko, eine definitive MS zu entwickeln [11, 22, 28]. Umgekehrt stellt der fehlende Nachweis oligoklonaler Banden bei Patienten mit untypischer klinischer Präsentation und fraglichen Entzündungsherden im MRT eine „red flag“ zum möglichen Vorliegen einer fehlerhaften MS-Diagnose dar [3, 23, 24]. Zusammenfassend sollte bei Patienten mit atypischer Präsentation oder fraglichen Befunden darauf geachtet werden, dass mehr als die minimalen Anforderungen der Diagnosekriterien erfüllt werden, dass bei Patienten mit Migräne und kardiovaskulären Risikofaktoren oligoklonale Banden vorliegen (und die Bestimmung bei initial negativen Befunden ggf. wiederholt wird), dass bei Patienten fortgeschrittenen Alters größere oder mindestens 6 mm große Entzündungsherde im MRT dargestellt werden können (zur Abgrenzung vaskulär bedingter Veränderungen) und dass ein regelmäßiges klinisch-radiologisches Monitoring erfolgt, um mögliche (sub-)klinische Demyelinisierungsepisoden zu detektieren und somit fehlerhafte MS-Diagnosen zu vermeiden [3, 23, 24].

Es konnte auch gezeigt werden, dass der Zugewinn an MS-Diagnosen mit einer Reduktion der Spezifität einhergeht. Die niedrigere diagnostische Spezifität der revidierten McDonald-Kriterien von 2017 war zu erwarten, da das Ziel der aktuellen Revision war, die Diagnose einer MS früher stellen zu können [28]. Exemplarisch hierfür ist der Einschluss der oligoklonalen Banden in die McDonald-Kriterien von 2017: Einerseits führt die hochgradig sensitive Detektion einer intrathekalen IgG-Synthese durch die oligoklonalen Banden zu einer erhöhten diagnostischen Sensitivität der McDonald-Kriterien von 2017 [32]. Andererseits stellen die oligoklonalen Banden einen unspezifischen Biomarker dar, der auch bei anderen entzündlichen neurologischen Erkrankungen vorkommen kann und somit mutmaßlich die erniedrigte diagnostische Spezifität der revidierten McDonald-Kriterien (59 % vs. 81 %) begründet [32]. Weiterhin ist beim Vergleich älterer, restriktiverer Diagnosekriterien mit neuen, eher inklusiven, in meist retrospektiven Studien erhobenen Diagnosekriterien eine niedrigere Spezifität zu erwarten. Außerdem können Studiendesign, kleine Studienpopulationen und kurzer Follow-up-Zeitraum zu einem Bias führen, der die Interpretation der jeweiligen Studienergebnisse beeinflusst. Die wenigen prospektiv durchgeführten Studien bestätigen diese Vermutung. In diesen ist die diagnostische Spezifität der McDonald-Kriterien von 2017 signifikant höher, verglichen mit der der retrospektiven Studien (82 % vs. 59 %, *p* = 0,0280; [1, 2, 4–10, 12, 13, 15, 16, 21, 25, 27, 29, 31, 33]). Ein weiterer Einflussfaktor, der die diagnostische Spezifität beeinflussen könnte, stellt der Einsatz von MS-Basistherapien während des Follow-ups von KIS-Patienten dar. Dieser könnte zu einer Verzögerung des Auftretens eines zweiten klinischen Schubs oder neuer Entzündungsherde führen, welche die verzögerte Diagnosestellung einer definitiven MS nach den McDonald-Kriterien von 2017 zur Folge hat. Belege hierfür finden sich in den Arbeiten von Pagani Cassara et al. und Hyun et al., in denen der Ausschluss von Patienten, welche ein MS-Basistherapeutikum erhalten haben, zu einer Steigerung der diagnostischen Spezifität von 54 % bzw. 53 % auf 88 % bzw. 85 % führte [10, 16].

Weiterhin stellt die Diagnosestellung einer MS bei Kindern eine besondere Herausforderung dar. Alle McDonald-Kriterien basieren auf Studien, welche das erste klinische Ereignis vereinbar mit einem MS-Schub bei Erwachsenen zwischen 18 und 50 Jahren in einer Population kaukasischstämmiger Patienten (Europa, USA, Kanada) beschreiben [4, 24]. Somit birgt die Diagnosestellung einer MS bei Patienten, die nicht diesem demografischen Profil entsprechen, ein erhöhtes Risiko für Fehldiagnosen [24]. Für die Revision der McDonald-Kriterien von 2010 konnte gezeigt werden, dass diese bei Erwachsenen und Kindern ähnlich gut anwendbar sind [4]. Problematisch stellte sich jedoch die Diagnosestellung bei Kindern unter 11 Jahren und mit multifokalen neurologischen Defiziten dar [4]. Drei der vorliegenden Studien untersuchten die Anwendung der revidierten McDonald-Kriterien von 2017 in Patientenkollektiven mit einem medianen Patientenalter zwischen 13,7 und 14,8 Jahren [4, 9, 31]. Hierbei zeigte sich, dass die berichtete diagnostische Sensitivität nicht wesentlich von der der übrigen Studien abwich (*p* = 0,7526).

## Konklusion

Ziel der Revision der McDonald-Kriterien von 2017 war es, die schnellere Diagnosestellung einer definitiven MS zu ermöglichen. Dieses Ziel wurde erreicht. Hauptsächlich aufgrund der Einführung der oligoklonalen Banden als Diagnosekriterium konnte der Anteil der MS-Diagnosen beim ersten klinischen Schubereignis erhöht und die Reduktion des Zeitraums zwischen erstem klinischem Schubereignis und Diagnosestellung verkürzt werden. Trotz der hohen Prävalenz der oligoklonalen Banden sollte bedacht werden, dass diese nicht spezifisch für die MS sind.
